# Traumatic Brain Injury Is Associated With Both Hemorrhagic Stroke and Ischemic Stroke: A Systematic Review and Meta-Analysis

**DOI:** 10.3389/fnins.2022.814684

**Published:** 2022-02-10

**Authors:** Donghao Qu, Wenchen Li, Shuyan Zhang, Ri Li, Haifeng Wang, Bo Chen

**Affiliations:** ^1^Department of Neurosurgery (Neurotrauma), The First Hospital of Jilin University, Changchun, China; ^2^Department of Library, The First Hospital of Jilin University, Changchun, China

**Keywords:** traumatic brain injury, hemorrhagic stroke, ischemic stroke, risk factor, meta-analysis

## Abstract

**Background:**

Traumatic brain injury (TBI) is considered a risk factor for the development of stroke (Hemorrhagic Stroke and Ischemic Stroke). We performed this systemic review and meta-analysis to determine the association of prior TBI with the subsequent diagnosis of stroke.

**Methods:**

We systematically searched PubMed, EMBASE, and the Cochrane Library for cohort studies involving TBI patients who subsequently developed stroke. Study selection, data extraction, and quality assessment were performed by two separate researchers. Data were analyzed with random-effects models, and a secondary analysis stratified by the type of stroke was performed.

**Results:**

Of the 741 identified studies, 6 studies were eligible for inclusion, with more than 2,200,000 participants. TBI predicted the occurrence of stroke in the random-effect model, with a relative risk of 2.14 (95% CI 1.97–2.32, *P* < 0.001). Furthermore, in the analysis of each type of stroke, TBI was associated with the incidence of ischemic stroke (RR 1.351 95% CI 1.212–1.506, *P* < 0.001), and TBI was associated with an even greater increase in the incidence of hemorrhagic stroke (RR 6.118 95% CI 5.265–7.108, *P* < 0.001).

**Conclusion:**

Our meta-analysis showed that TBI was associated with a more than two-fold increase in the risk of stroke. However, owing to the high degree of heterogeneity, decisions should be made on a patient-by-patient basis. The occurrence of TBI is associated with the development of both hemorrhagic and ischemic stroke, and the risk of hemorrhagic stroke is much higher than that of ischemic stroke in TBI patients.

## Introduction

Stroke is the second most common cause of death (Wang et al., 2017) and the third most common cause of disability (Murray et al., 2012) worldwide. The primary causes of stroke are hypertension, diabetes, hypercholesterolemia, coronary artery disease, atrial fibrillation, sedentary lifestyle, psychosocial stress, and depression (O'Donnell et al., 2010). Identifying subclinical stroke risk factors may enable us to implement early and potentially more effective stroke prevention measures. Some recent studies on traumatic brain injury (TBI) investigated whether TBI is another potential stroke risk factor. TBI is a major cause of morbidity and mortality and has been described as a chronic, evolving, and perhaps lifelong disorder because of the associated long-term problems or impairments. TBI survivors may suffer long-term physical, cognitive, and psychological disorders (Stocchetti and Zanier, 2016), all of which can affect the long-term outcomes. In recent years, the research focus has mainly been on the relationships of TBI with neurologic diseases and psychiatric diseases (Perry et al., 2016; Wilson et al., 2017). However, both stroke and TBI are very common and are responsible for substantial social and economic burdens. In 2011, a study (Chen et al., 2011) formally suggested a clear link between TBI and subsequent stroke for the first time, but very few studies have been performed to validate and assess this link, including its directionality and internal linkage.

It is important to identify potential risk factors, but mechanistic research is difficult, mainly because of a lack of understanding of the pathophysiology. Previous studies hypothesized that vascular damage from TBI results in the disturbance of the blood supply to the brain, which may induce stroke in survivors (Chen et al., 2011). Stroke can be classified as ischemic or hemorrhagic. Some studies investigated ischemic stroke, and some investigated hemorrhagic stroke. However, few studies have investigated all subtypes of stroke after TBI. In addition, relying on a single study result in wide confidence intervals for the risk estimates. Therefore, there is an urgent need for further research. Meta-analyses of existing datasets make it possible to elucidate the associations between TBI and ischemic and hemorrhagic stroke and to summarize and make inferences from the wider clinical picture based on the available evidence. Accordingly, we performed a systematic review and meta-analysis to explore whether the occurrence of TBI leads to an increased risk of stroke; if such an association exists, then there is a new target for the treatment of posttraumatic stroke. Furthermore, this analysis is expected to provide insights into similar issues in other types of TBI/neuropathology.

## Methods

This meta-analysis followed the Meta-analyses Of Observational Studies in Epidemiology (MOOSE) guidelines (Stroup et al., 2000) and the Preferred Reporting Items for Systematic Reviews and Meta-Analyses (PRISMA) statement (Liberati et al., 2009).

### Search Strategy and Selection Criteria

**Figure 1:**
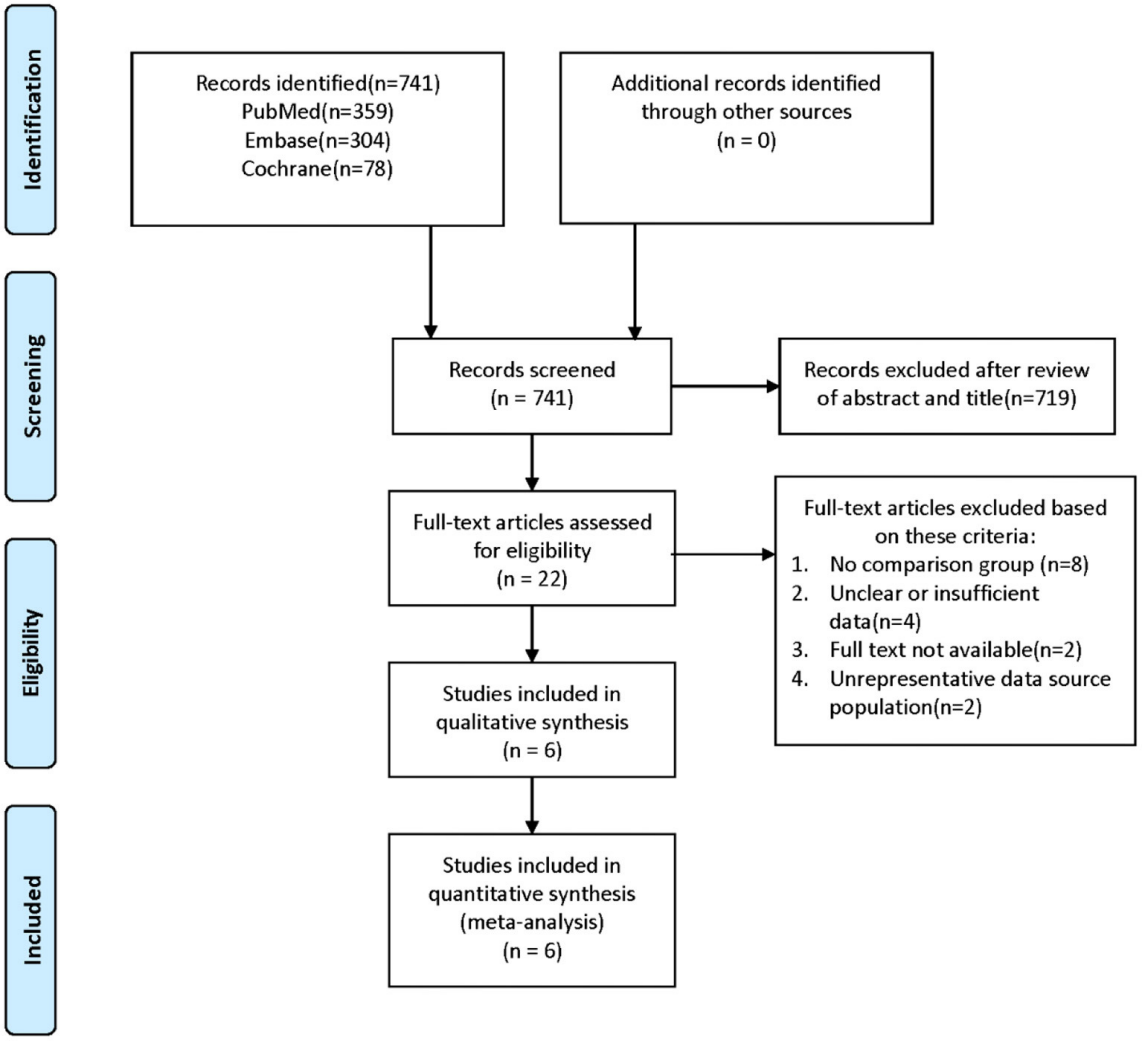
Flow chart of the literature search.

A researcher conducted a comprehensive search in PubMed, EMBASE, and the Cochrane Library. The first search was conducted in PubMed using a comprehensive strategy. We used three components in each search: component A identified papers with the keywords “Traumatic Brain Injuries,” “Brain Trauma,” “Traumatic Encephalopathies,” and “TBI.” Component B identified papers with the other keywords “Stroke,” “Cerebrovascular Accident,” “Cerebrovascular Apoplexy,” “Brain Vascular Accident,” “Cerebrovascular Stroke,” “Apoplexy,” “Cerebral Stroke,” “Acute Stroke,” and “Acute Cerebrovascular Accident.” Component C was used to define the design of the study as a “cohort study” and “cohort analysis.” Finally, we combined component A, component B, and component C with the operator “and.” The subject headings and keywords used were adapted to the other databases. The search was performed up to December 30, 2020 (See Figure 1 for more details on the retrieval strategy).

We applied the following inclusion criteria to select papers for further review: (1) the study included adult subjects older than 18 years of age at the time of evaluation, (2) the study included at least 100 subjects, (3) the study included patients with TBI before the diagnosis of stroke (patients who had a diagnosis of acute stroke simultaneously during the same admission were excluded), and (4) the study had a sufficient follow-up duration.

### Data Extraction and Quality Assessment

Two authors screened all titles and abstracts of the studies identified by our initial search and determined which of the studies should be assessed further. The full texts of the relevant articles were retrieved and screened independently by the same two authors. The necessary data were extracted. The extracted data included the study characteristics (author, publication year, country, design, and sample size), patient characteristics (demographics and history of diabetes mellitus, chronic kidney disease, atrial fibrillation, coronary artery disease, hyperlipidemia, and hypertension), and outcomes and results (TBI status, follow-up duration, and the number of strokes, including all strokes, ischemic strokes, and hemorrhagic strokes, that occurred during follow-up in both the TBI group and the control group).

The quality of the included studies was assessed by two separate researchers, and any disagreement was resolved through discussion and consensus. For the six included cohort studies, we used the Newcastle-Ottawa Scale (NOS, **Table 2**) (Stang, 2010) to assess the risk of bias in the following domains: selection (representativeness of the exposed cohort, selection of the non-exposed cohort, ascertainment of exposure, demonstration that the outcome of interest was not present at the start of the study), comparability (comparability of cohorts based on the design or analysis), and outcome (assessment of outcome, sufficient follow-up for the outcomes to occur, adequacy of the follow-up of cohorts).

### Statistical Analysis

The meta-analysis was performed using dichotomous variables (comparing the TBI and stroke group, the TBI and no stroke group, the no TBI and stroke group, and the no TBI and no stroke group) extracted from the qualified studies. The meta-analysis was performed with Stata 12.0. Given the differences in the study design, subject characteristics, severity of TBI, sample size, and subtypes of stroke, data were pooled and evaluated with a random-effects model. Forest plots were generated to display the individual study relative risk (RR) and 95% confidence interval (95% CI). We used the inconsistency index test (*I*^2^ statistic) to assess the heterogeneity among the studies. To assess the presence of publication bias, we used the Begg and Mazumdar rank correlation test. All *p* < 0.05 were considered statistically significant.

## Results

### Study Selection and Study Characteristics

After screening the titles and abstracts of 741 studies and reading the full texts of 22 studies, we identified 6 articles (Chen et al., 2011; Burke et al., 2013; Lee et al., 2014; Liao et al., 2014; Albrecht et al., 2015; Eric Nyam et al., 2019) that met all the inclusion criteria for this meta-analysis. The study selection steps are summarized in Figure 1. In total, 548,046 TBI patients and 1,696,149 control patients were included in the study. Therefore, the sample size was sufficient to perform a meta-analysis of all strokes, ischemic strokes, and hemorrhagic strokes. Four studies were from Taiwan, China (Chen et al., 2011; Lee et al., 2014; Liao et al., 2014; Eric Nyam et al., 2019), and two studies were from the United States (Burke et al., 2013; Albrecht et al., 2015). Of all the included studies, only one was a prospective cohort study (Chen et al., 2011), and the rest were retrospective cohort studies (Burke et al., 2013; Lee et al., 2014; Liao et al., 2014; Albrecht et al., 2015; Eric Nyam et al., 2019). Five studies focused on middle-aged people (mean age 41.6~50.3 years) (Chen et al., 2011; Burke et al., 2013; Lee et al., 2014; Liao et al., 2014; Eric Nyam et al., 2019), while one study evaluated the relationship between TBI and stroke in older patients (mean age 81.0 years) (Albrecht et al., 2015). In terms of TBI severity, all except one focused on mild TBI (Lee et al., 2014), and in this article, patients with skull fractures were excluded. In four studies (Chen et al., 2011; Liao et al., 2014; Albrecht et al., 2015; Eric Nyam et al., 2019), stroke was defined as ischemic stroke, hemorrhage stroke, and unspecified stroke, but only three studies (Chen et al., 2011; Albrecht et al., 2015; Eric Nyam et al., 2019) reported detailed data (the number of stroke patients, ischemic stroke patients, and hemorrhage stroke patients). The remaining two studies (Burke et al., 2013; Lee et al., 2014) focused on the relationship between TBI and ischemic stroke. Detailed characteristics of the included studies are presented in Table 1.

**Table 1:** Main characteristics of studies included in the meta-analysis.

**Study number**	**References**	**Study design**	**County**	**Follow-up**	**TBI status**	**Number of**	**Age**	**Women, no**	**Hypertension**	**Diabetes**	**Chronic**	**Atrial**	**Coronary**	**Hyperlipidemia**
						**subjects**	**(years, SD)**	**(% female)**		**mellitus**	**kidney**	**fibrillation**	**artery**	
										**(%)**	**disease**	**(%)**	**disease**	
											**(%)**		**(%)**	
1	Chen et al. (2011)	Prospective cohort	Taiwan, China	5 years	TBI	23,199	41.6 (18.4)	10,768 (46.4)	3,802 (16.4)	2,037 (8.8)	No data	99 (0.4)	1,808 (7.8)	1,767 (7.6)
					No TBI	69,597		32,304 (46.4)	9,916 (14.3)	4,836 (7.0)	No data	199 (0.3)	4,344 (6.2)	5,342 (7.7)
2	Burke et al. (2013)	Retrospective cohort	United States	2 years	TBI	436,630	49.2 (22.4)	204,298 (46.8)	75,438 (17.3)	31,897 (7.3)	7386 (1.7)	12,359 (2.8)	16,573 (3.8)	20,873 (4.8)
					No TBI	736,723	50.3 (20.1)	363,210 (49.3)	125,752 (17.1)	59,141 (8.0)	13,538 (1.8)	14,702 (2.0)	24,930 (3.4)	41,206 (5.6)
3	Liao et al. (2014)	Retrospective cohort	Taiwan, China	No data	TBI	30,165	44.5 (17.8)	14,936 (49.6)	7,398 (24.5)	3,592 (11.9)	353 (1.2)	No data	No data	3,826 (12.7)
					No TBI	120,660	43.9 (17.3)	59,852 (49.6)	26,592 (22.0)	10,826 (9.0)	799 (0.7)	No data	No data	13,906 (11.5)
4	Lee et al. (2014)	Retrospective cohort	Taiwan, China	3.88 (0.55)	TBI	24,905	46.1 (20.1)	13,091 (52.6)	4,936 (19.8)	2,466 (9.9)	No data	133 (0.5)	3,878 (15.6)	2,849 (11.4)
				1.94 (1.18)	No TBI	719,811	43.5 (16.3)	370,830 (51.5)	111,872 (15.5)	51,555 (7.2)	No data	2,654 (0.4)	78,417 (10.9)	71,010 (9.9)
5	Albrecht et al. (2015)	Retrospective cohort	United States	No data	TBI status known	16,936	81.0 (7.9)	10,557 (6.2)	No data	6,535 (39)	No data	4,664 (28)	No data	No data
6	Eric Nyam et al. (2019)	Retrospective cohort	Taiwan, China	5 years	TBI	16,211	No data	6,382 (39.37)	2,467 (15.22)	1,339 (8.26)	250 (1.54)	No data	No data	No data
					No TBI	32,422	No data	12,746 (39.31)	5,173 (15.96)	2,577 (7.95)	558 (1.72)	No data	No data	No data

*TBI, traumatic brain injury; SD, standard deviation*.

### Assessment of the Quality of the Included Studies

The results of the quality assessment are presented in Table 2. All included studies were assessed by two investigators, and any disagreements were resolved through discussion and joint assessment until a consensus was reached. All but two studies declared that follow-up continued for each participant until the diagnosis of stroke or until censoring because of death. Only one study used a single cohort in which the outcome was measured in the same study population before and after the occurrence of TBI, a design that made it impossible to compare cases and controls.

**Table 2:** Risk of bias assessment for cohort studies (based on modified Newcastle-Ottawa scale) on stroke in patients with TBI.

**References**	**Selection**	**Comparability**	**Outcome**	**Total score**
Chen et al. (2011)	****	***	***	10/10
Burke et al. (2013)	****	***	**	9/10
Liao et al. (2014)	****	***	***	10/10
Lee et al. (2014)	****	***	***	10/10
Albrecht et al. (2015)	***	–	***	6/10
Eric Nyam et al. (2019)	****	***	**	9/10

A study can be given a maximum score of 10 stars (^*^). Lower total score means a higher risk of bias.

### Association Between TBI and Stroke

**Figure 2:**
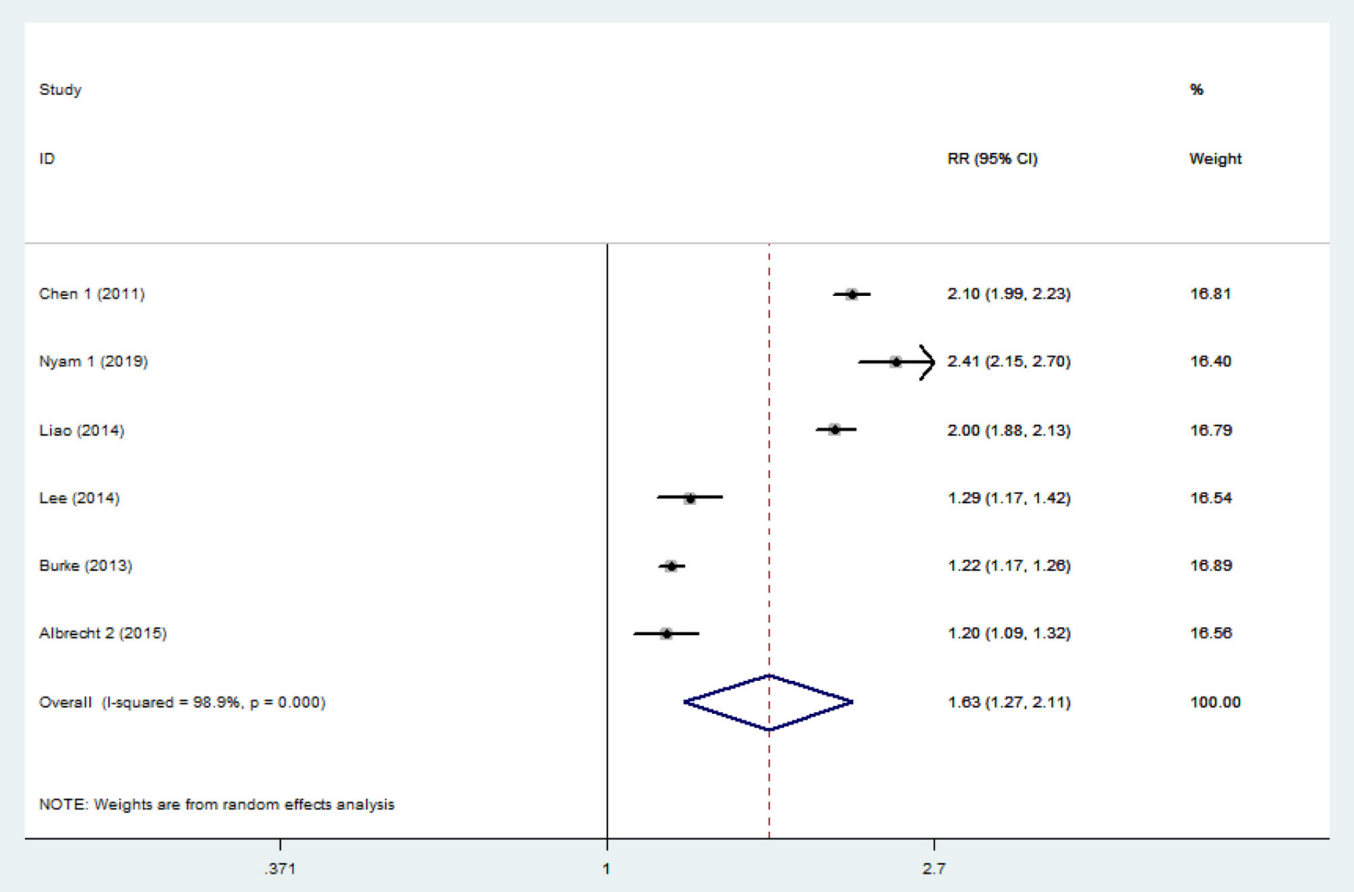
Forest plots of the association between traumatic brain injury and future stroke in all included studies. The meta-analysis was performed using a random-effects model. The overall analysis is shown. Diamonds represent the pooled estimates, and the horizontal lines represent the 95% confidence intervals. RR, relative risk; CI, confidence interval.

**Figure 3:**
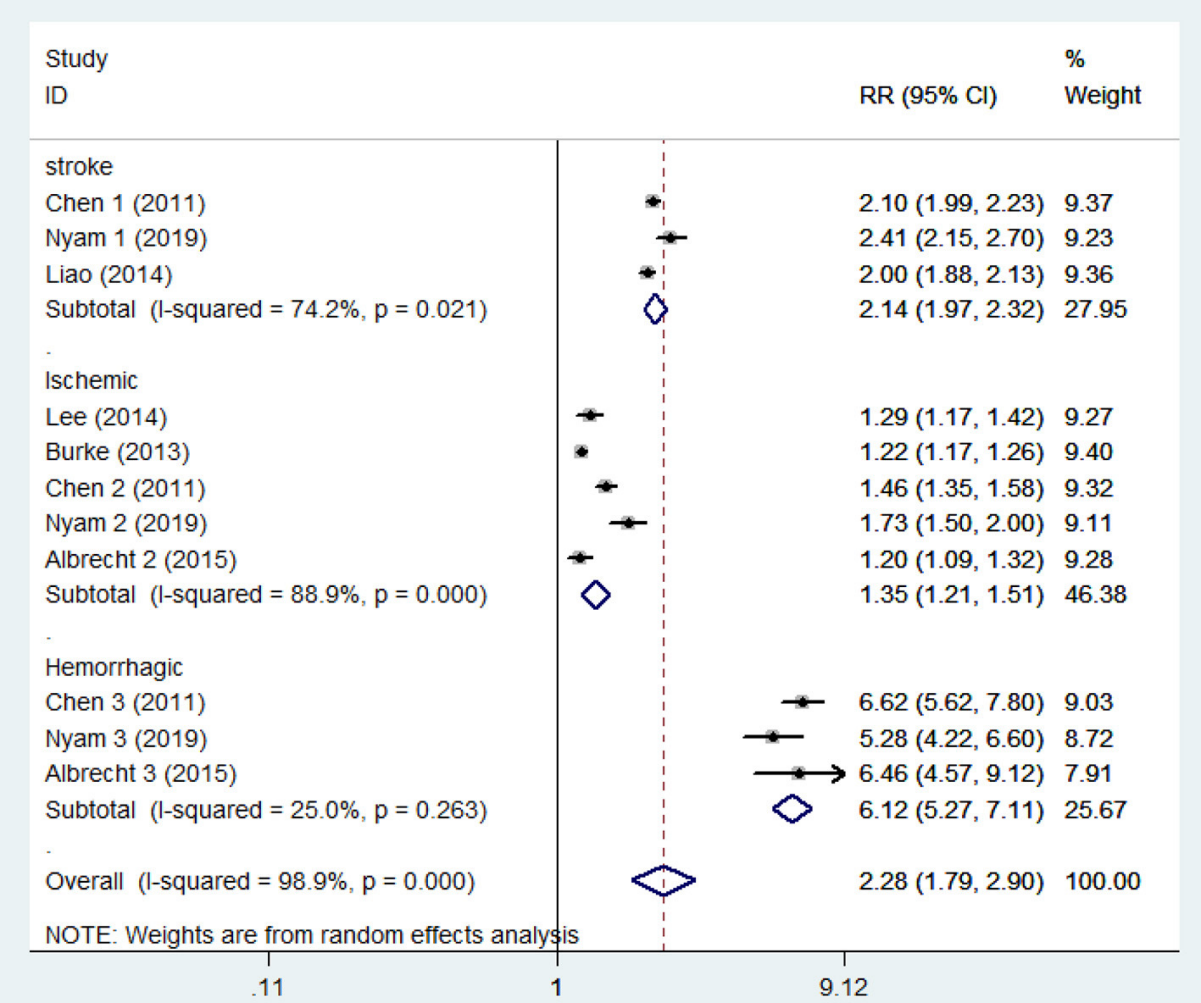
Forest plots of the association between traumatic brain injury and future stroke in all included studies. The meta-analysis was performed using a random-effects model. The subgroup analysis is shown. Diamonds represent the pooled estimates, and the horizontal lines represent the 95% confidence intervals. RR, relative risk; CI, confidence interval.

The pooled analysis of the 6 studies showed that the patients who had a TBI had a greater chance of stroke, with a random-effect relative risk (RR) of 1.634 (95% CI 1.266~2.109, *P* < 0.001, Figure 2). However, there was statistically significant heterogeneity among the studies according to all the detection methods (Figure 2). In the sensitivity analysis of the six studies, the results remained consistent (Supplementary Figure 1). There was no significant publication bias according to Begg's test (*P* > 0.05). Three of the six included studies included both hemorrhagic and ischemic stroke patients, while the other three studies included only patients with ischemic stroke. Thus, the heterogeneity among studies is mainly due to differences in stroke subtypes, in addition to differences in demographic characteristics. Subgroup analysis was performed to not only reveal the relationships between TBI and the different subtypes of stroke but also effectively reduce the heterogeneity. The subgroup analysis revealed a strong correlation between TBI and stroke (RR = 2.14 95% CI 1.97–2.32, *P* < 0.001). Furthermore, based on data from three studies involving 158,365 individuals, the risk of hemorrhagic stroke in patients with TBI was ~6-fold higher than that in patients without TBI (RR 6.12 95% CI 5.265–7.108, *P* < 0.001). There was neither significant heterogeneity (Figure 3) nor significant publication bias according to Begg's test (*P* > 0.05). In the subgroup analysis of 5 studies involving 2,076,343 individuals, the risk of ischemic stroke was higher in patients with TBI than in those without TBI (RR 1.35 95% CI 1.212–1.506, *P* < 0.001). However, these findings should be interpreted with caution given the large degree of heterogeneity. In the sensitivity analysis of the five studies, the occurrence of TBI was associated with a higher incidence of ischemic stroke (Supplementary Figure 2). There was no significant publication bias according to Begg's test (*P* > 0.05).

## Discussion

In this systematic review and meta-analysis involving over 2,200,000 patients from 6 studies, the results demonstrated that in addition to traditional risk factors, the occurrence of TBI was associated with an increased incidence of stroke. Ischemic and hemorrhagic stroke are significantly associated with prior TBI. The magnitudes of the effect of TBI on different types of stroke were comparable, with a higher RR for hemorrhagic stroke. These results support the identification of TBI as an independent risk factor for stroke and could guide prognostic prediction and stroke prevention in patients with TBI.

Both TBI and stroke are important conditions. Patients with TBI or stroke and their family members have to cope with a disability, mobility issues, psychological trauma, and even economic pressure during the long period of acute and chronic rehabilitation. A proportion of strokes can neither be explained by the frequently used stroke prediction models nor by known risk factors, so we hypothesized that there might be some unknown risk factors for stroke involving as yet undetermined mechanisms. Numerous studies have demonstrated that multiple chronic diseases can be attributed to previous TBI, including mild cognitive impairment (Moretti et al., 2012), neurodegenerative diseases (Crane et al., 2016; Perry et al., 2016), posttraumatic epilepsy (Vaaramo et al., 2014), neuroendocrine disorders (Behan et al., 2008), psychiatric illness (Perry et al., 2016), obstructive sleep apnea (Castriotta et al., 2007), and metabolic dysfunction (Børsheim et al., 2007). Considering the complex pathological mechanism underlying TBI (VanItallie, 2019) and the close relationships of TBI with the abovementioned diseases, we suggest that there are several possible explanations for the association between TBI and stroke. After experiencing trauma, multiple pathological alterations may occur in the brain, including hemodynamic derangements (Steinman et al., 2019), alterations of the coagulation system (Maegele et al., 2017), disturbances of cerebral oxygenation, and metabolism and vascular deformities. Shock and systemic inflammation are common acute reactions after TBI, and the link between inflammation and coagulation is enhanced *via* the complement system and monocytes (Atefi et al., 2016; Foley and Conway, 2016). Moreover, both vascular regeneration and disturbances in blood flow are important long-term reactions (Kenney et al., 2016), which trigger the interaction between platelets and the subendothelial matrix (Steinman et al., 2019). The processes may not only occur simultaneously but also be interconnected, and they modify the coagulation balance. This alters the clotting balance, leading to thrombosis (Maegele et al., 2017), which could contribute to the development of ischemic stroke. The excessive consumption of platelets and coagulation factors result in an increased risk of bleeding, which may contribute to the development of hemorrhagic stroke. Some previous studies found other risk factors that may be associated with the occurrence of posttraumatic stroke. Based on credible studies, patients with TBI are more likely to suffer from neurologic and psychiatric diseases, including migraine, epilepsy, and schizophrenia, which are known risk factors for stroke (Perry et al., 2016). The use of atypical antipsychotic drugs has also been found to be a risk factor for stroke (Gill et al., 2005; Taylor et al., 2019). Due to the high rate of disability, a large proportion of people with TBI suffer from physical activity limitations, which constitute another risk factor for stroke (Kyu et al., 2016; Kramer et al., 2019).

Based on several authoritative clinical studies (Chen et al., 2011; Burke et al., 2013; Lee et al., 2014; Liao et al., 2014; Albrecht et al., 2015; Kowalski et al., 2017; Eric Nyam et al., 2019) and our meta-analysis, the conclusion that TBI is a new risk factor for stroke is clear. Therefore, targeted measures are necessary. Previous studies suggested that early, timely, and effective treatments are associated with positive outcomes of posttraumatic stroke due to the narrow window for treatment (Khandelwal et al., 2016). When the initial stroke symptoms occur, patients and their families need to be aware of the danger and promptly seek medical care, which is not something that happens frequently due to the lack of health awareness and relevant medical knowledge among the general population. Conventional coagulation assays (CCAs) remain the most commonly used method of assessing blood coagulation after TBI (Joseph et al., 2014). Some recent studies have suggested that global hemostatic assays, such as thromboelastography (TEG), rotational thromboelastometry (ROTEM), and thrombin generation tests, are better assessments, but they are currently only used for experimental research (Hunt et al., 2015). Overall, rapid and reliable coagulation information is provided by global hemostatic assays, and more importantly, the results can be used to guide treatment (Hagemo et al., 2013; Whiting and DiNardo, 2014). To better understand the specific status of the cerebrovasculature after stroke, patients should also undergo magnetic resonance imaging (Thomalla et al., 2017), angiography, or other specialized neuroimaging. If abnormal clot formation dynamics or vascular dissection, both of which are important aspects of the possible mechanism, are identified on the relevant imaging examinations, the medical team should be especially cautious. Because a stroke can occur at any time and it is difficult to assess the exact time of stroke onset, we recommend that survivors of TBI with the above imaging abnormalities or risk factors should be promptly treated with surgical or pharmacological therapy, depending on their actual condition. Furthermore, according to our meta-analysis, the risk of hemorrhagic stroke is higher than that of ischemic stroke in TBI survivors, which should be taken into account by the treatment team when developing a treatment plan. If effective clinical prediction models are developed for post-traumatic hemorrhagic stroke and ischemic stroke, respectively, highly targeted and effective treatment protocols, including the correction of coagulation disorders, can be specified for the different types of strokes according to the current clinical practice guidelines (Spahn et al., 2019), potentially leading to better outcomes for patients with TBI. Secondary prevention, such as smoking cessation, proper nutrition, control of hypertension, physical activity, and maintenance of a healthy psychological status, is also important in the rehabilitation stage and for the rest of the patient's life.

There are some limitations of our study. First, some of the studies did not provide specific data regarding the follow-up duration. However, some of the original texts specifically reported the follow-up duration or stated that adequate follow-up was conducted for each participant. Second, the included studies did not report the clinical features (the classification of the severity of TBI, imaging information, the cause of the TBI, the location of the stroke). The lack of the above information constrained the subgroup analyses and prevented the further analysis of the risk factors for and mechanisms of posttraumatic stroke. It will be important to include those factors in future studies; however, the available data were sufficient to preliminarily conclude that there is a relationship between stroke and TBI. Third, the control groups in the studies included were from different populations; for example, the control group was composed of those who had previously visited ambulatory care centers or were hospitalized for reasons other than TBI, the control group was composed of patients who had a fracture in a location other than the head and neck, and the control group was composed of patients aged ≥65 years. The source of the control group contributed to the heterogeneity to a certain extent. However, these small differences between studies seem unlikely to have affected our overall conclusions. Fourth, most of the data came from hospital admissions or Medicare records. A portion of patients with mild TBI do not visit the hospital, which would lead to a relative increase in the number of patients with stroke in the control group and result in an underestimation of the correlation between TBI and stroke. Finally, hemorrhagic stroke may be a sequela of TBI. It is relatively easy for clinicians to distinguish between intracerebral hemorrhage and traumatic brain hemorrhage, although errors are possible.

Globally, the effect of gender differences on stroke risk in the general population is not significant, but in low- and middle-income countries, the risk of stroke increases due to increased exposure of men to risk factors, such as smoking, lack of exercise, environmental pollution, as well as metabolic diseases, like high blood sugar, high cholesterol (Feigin et al., 2016). Therefore, differences in the gender composition of the TBI and control groups need to be taken into account in our meta-analysis. There are a total of six original studies were included in our meta-analysis study. Five of them (Chen et al., 2011; Burke et al., 2013; Liao et al., 2014; Albrecht et al., 2015; Eric Nyam et al., 2019) have shown that the gender distribution is balanced rather than statistically different (*P* > 0.05) in TBI vs. controls, and there was only one study (Lee et al., 2014) of an imbalance in gender distribution between the two groups. Therefore, we did a sensitivity analysis on gender distribution by excluding the one study with an unbalanced gender factor, and performed data synthesis for the remaining five studies in the meta-analysis, and found that the RR did not change significantly from before exclusion (RR = 1.63 95% CI 1.27–2.11) to after exclusion (R = 1.71 95% CI 1.28–2.30), which proved that our results were stable and the gender did not affect the final results of our meta-analysis. Similarly, we performed sensitivity analysis on the results of subgroup analysis. We found that the change in RR was acceptable in the ischemic group, from before exclusion (RR = 1.35 95% CI 1.21–1.51) to after exclusion (R = 1.37 95% CI 1.19–1.57), which indicated that the gender factor did not influence the results of subgroup analysis. The study on the correlation between sex and stroke onset after TBI is valuable, but unfortunately, our meta-analysis is limited in this aspect, and we hope there will be enough reliable studies in this direction in the future.

Our study aimed to systematically assess the relationship between TBI and subsequent stroke, and an understanding of this relationship can accurately and effectively guide clinical treatment. The strength of this meta-analysis was the inclusion of patients with a variety of different types of stroke rather than those with a single type. Due to the completely different treatments used, the distinction between ischemic stroke and hemorrhagic stroke has substantial clinical significance. Most importantly, these results can contribute to an improvement in the management of TBI patients. Specifically, awareness and vigilance about recognizing stroke-related symptoms, the timely performance of laboratory and imaging tests, and the initiation of surgical or pharmacologic treatments according to the guidelines can lead to improved outcomes, reduce the incidence of stroke, and minimize the loss of life and the development of serious health problems.

## Conclusion

In conclusion, this systematic review and meta-analysis showed that TBI is significantly associated with an increased risk of all types of stroke, including ischemic stroke and hemorrhagic stroke, and that the association between TBI and hemorrhagic stroke was particularly strong. Because of the heterogeneity among the included studies and the limitations on further analyses, more studies with uniform assessments and larger samples sizes are needed to confirm our results. Moreover, it is necessary to investigate the potential mechanisms underlying stroke evolution after TBI and the 3 effectiveness of preventive measures in the future.

## Data Availability Statement

The original contributions presented in the study are included in the article/Supplementary Material; further inquiries can be directed to the corresponding authors.

## Author Contributions

DQ and WL: literature search, data extraction, and drafting of the manuscript. DQ and RL: statistical analysis. SZ and DQ: quality evaluation. BC and HW: design of the study and commentary on important intellectual content. All authors contributed to the article and approved the submitted version.

## Funding

This study was supported by the National Natural Science Foundation of China (81871555), the National Natural Science Foundation of China (81701641), Natural Science Foundation of Jilin Province (20210101345JC), the Foundation of Jilin Finance Department (2018SCZWSZX-006), the Foundation of Jilin Finance Department (JLSCZD2019-006), the Foundation of Jilin Finance Department (2018SCZWSZX-040), and the Translational-Clinical Joint Fund of the First Hospital of Jilin University (2018-ZL-11).

## Conflict of Interest

The authors declare that the research was conducted in the absence of any commercial or financial relationships that could be construed as a potential conflict of interest.

## Publisher's Note

All claims expressed in this article are solely those of the authors and do not necessarily represent those of their affiliated organizations, or those of the publisher, the editors and the reviewers. Any product that may be evaluated in this article, or claim that may be made by its manufacturer, is not guaranteed or endorsed by the publisher.

## Supplementary Material

Supplementary Figure 1 Sensitivity analysis on gender. **(A)** Forest plots of the association between traumatic brain injury and future stroke in all included studies. **(B)** Forest plots of the association between traumatic brain injury and future stroke after excluding the study that had an unbalanced distribution of gender between TBI and control groups. Diamonds represent the pooled estimates, and the horizontal lines represent the 95% confidence intervals. RR, relative risk; CI, confidence interval.

Supplementary Figure 2 Gender sensitivity analysis in subgroup analysis. **(A)** Forest plots of subgroup analysis of the association between traumatic brain injury and all types of stroke in all included studies. **(B)** Forest plots of subgroup analysis of the association between traumatic brain injury and all types of stroke after excluding the study that had an unbalanced distribution of gender between TBI and control groups. Diamonds represent the pooled estimates, and the horizontal lines represent the 95% confidence intervals. RR, relative risk; CI, confidence interval.

## References

[B1] Albrecht J. S. Liu X. Smith G. S. Baumgarten M. Rattinger G. B. Gambert S. R. . (2015). Stroke incidence following traumatic brain injury in older adults. J. Head Trauma Rehabil. 30, E62–E67. 10.1097/HTR.0000000000000035 24816156 PMC4524572

[B2] Atefi G. Aisiku O. Shapiro N. Hauser C. Dalle Lucca J. Flaumenhaft R. . (2016). Complement activation in trauma patients alters platelet function. Shock 46(3 Suppl 1), 83–88. 10.1097/SHK.0000000000000675 27355402

[B3] Behan L. A. Phillips J. Thompson C. J. Agha A. (2008). Neuroendocrine disorders after traumatic brain injury. J. Neurol. Neurosurg. Psychiatry 79, 753–759. 10.1136/jnnp.2007.132837 18559460

[B4] Børsheim E. Bui Q. U. Wolfe R. R. (2007). Plasma amino acid concentrations during late rehabilitation in patients with traumatic brain injury. Arch. Phys. Med. Rehabil. 88, 234–238. 10.1016/j.apmr.2006.11.003 17270522

[B5] Burke J. F. Stulc J. L. Skolarus L. E. Sears E. D. Zahuranec D. B. Morgenstern L. B. (2013). Traumatic brain injury may be an independent risk factor for stroke. Neurology 81, 33–39. 10.1212/WNL.0b013e318297eecf 23803315 PMC3770205

[B6] Castriotta R. J. Wilde M. C. Lai J. M. Atanasov S. Masel B. E. Kuna S. T. (2007). Prevalence and consequences of sleep disorders in traumatic brain injury. J. Clin. Sleep Med. 3, 349–356. 10.5664/jcsm.26855 17694722 PMC1978308

[B7] Chen Y. H. Kang J. H. Lin H. C. (2011). Patients with traumatic brain injury: population-based study suggests increased risk of stroke. Stroke 42, 2733–2739. 10.1161/STROKEAHA.111.620112 21799162

[B8] Crane P. K. Gibbons L. E. Dams-O'Connor K. Trittschuh E. Leverenz J. B. Keene C. D. . (2016). Association of traumatic brain injury with late-life neurodegenerative conditions and neuropathologic findings. JAMA Neurol. 73, 1062–1069. 10.1001/jamaneurol.2016.1948 27400367 PMC5319642

[B9] Eric Nyam T. T. Ho C. H. Chio C. C. Lim S. W. Wang J. J. Chang C. H. . (2019). Traumatic brain injury increases the risk of major adverse cardiovascular and cerebrovascular events: a 13-year, population-based study. World Neurosurg. 122, e740–e753. 10.1016/j.wneu.2018.10.130 30391613

[B10] Feigin V. L. Roth G. A. Naghavi M. Parmar P. Krishnamurthi R. Chugh S. . (2016). Global burden of stroke and risk factors in 188 countries, during 1990-2013: a systematic analysis for the Global Burden of Disease Study 2013. Lancet Neurol. 15, 913–924. 10.1016/S1474-4422(16)30073-4 27291521

[B11] Foley J. H. Conway E. M. (2016). Cross talk pathways between coagulation and inflammation. Circ. Res. 118, 1392–1408. 10.1161/CIRCRESAHA.116.306853 27126649

[B12] Gill S. S. Rochon P. A. Herrmann N. Lee P. E. Sykora K. Gunraj N. . (2005). Atypical antipsychotic drugs and risk of ischaemic stroke: population based retrospective cohort study. BMJ 330:445. 10.1136/bmj.38330.470486.8F 15668211 PMC549652

[B13] Hagemo J. S. Næss P. A. Johansson P. Windeløv N. A. Cohen M. J. Røislien J. . (2013). Evaluation of TEG(®) and RoTEM(®) inter-changeability in trauma patients. Injury 44, 600–605. 10.1016/j.injury.2012.11.016 23260867

[B14] Hunt H. Stanworth S. Curry N. Woolley T. Cooper C. Ukoumunne O. . (2015). Thromboelastography (TEG) and rotational thromboelastometry (ROTEM) for trauma induced coagulopathy in adult trauma patients with bleeding. Cochrane Datab. Syst. Rev. 2015:Cd010438. 10.1002/14651858.CD010438.pub2 25686465 PMC7083579

[B15] Joseph B. Aziz H. Zangbar B. Kulvatunyou N. Pandit V. O'Keeffe T. . (2014). Acquired coagulopathy of traumatic brain injury defined by routine laboratory tests: which laboratory values matter? J. Trauma Acute Care Surg. 76, 121–125. 10.1097/TA.0b013e3182a9cc95 24368366

[B16] Kenney K. Amyot F. Haber M. Pronger A. Bogoslovsky T. Moore C. . (2016). Cerebral vascular injury in traumatic brain injury. Exp. Neurol. 275(Pt 3), 353–366. 10.1016/j.expneurol.2015.05.019 26048614

[B17] Khandelwal P. Yavagal D. R. Sacco R. L. (2016). Acute ischemic stroke intervention. J. Am. Coll. Cardiol. 67, 2631–2644. 10.1016/j.jacc.2016.03.555 27256835

[B18] Kowalski R. G. Haarbauer-Krupa J. K. Bell J. M. Corrigan J. D. Hammond F. M. Torbey M. T. . (2017). Acute ischemic stroke after moderate to severe traumatic brain injury: incidence and impact on outcome. Stroke 48, 1802–1809. 10.1161/STROKEAHA.117.017327 28611087 PMC6025795

[B19] Kramer S. F. Hung S. H. Brodtmann A. (2019). The impact of physical activity before and after stroke on stroke risk and recovery: a narrative review. Curr. Neurol. Neurosci. Rep. 19:28. 10.1007/s11910-019-0949-4 31011851

[B20] Kyu H. H. Bachman V. F. Alexander L. T. Mumford J. E. Afshin A. Estep K. . (2016). Physical activity and risk of breast cancer, colon cancer, diabetes, ischemic heart disease, and ischemic stroke events: systematic review and dose-response meta-analysis for the Global Burden of Disease Study 2013. BMJ 354:i3857. 10.1136/bmj.i3857 27510511 PMC4979358

[B21] Lee Y. K. Lee C. W. Huang M. Y. Hsu C. Y. Su Y. C. (2014). Increased risk of ischemic stroke in patients with mild traumatic brain injury: a nationwide cohort study. Scand. J. Trauma Resusc. Emerg. Med. 22:66. 10.1186/s13049-014-0066-y 25406859 PMC4239396

[B22] Liao C. C. Chou Y. C. Yeh C. C. Hu C. J. Chiu W. T. Chen T. L. (2014). Stroke risk and outcomes in patients with traumatic brain injury: 2 nationwide studies. Mayo Clin. Proc. 89, 163–172. 10.1016/j.mayocp.2013.09.019 24485130

[B23] Liberati A. Altman D. G. Tetzlaff J. Mulrow C. Gøtzsche P. C. Ioannidis J. P. . (2009). The PRISMA statement for reporting systematic reviews and meta-analyses of studies that evaluate health care interventions: explanation and elaboration. Ann. Intern. Med. 151, W65–94. 10.7326/0003-4819-151-4-200908180-00136 19622512

[B24] Maegele M. Schöchl H. Menovsky T. Maréchal H. Marklund N. Buki A. . (2017). Coagulopathy and haemorrhagic progression in traumatic brain injury: advances in mechanisms, diagnosis, and management. Lancet Neurol. 16, 630–647. 10.1016/S1474-4422(17)30197-7 28721927

[B25] Moretti L. Cristofori I. Weaver S. M. Chau A. Portelli J. N. Grafman J. (2012). Cognitive decline in older adults with a history of traumatic brain injury. Lancet Neurol. 11, 1103–1112. 10.1016/S1474-4422(12)70226-0 23153408

[B26] Murray C. J. Vos T. Lozano R. Naghavi M. Flaxman A. D. Michaud C. . (2012). Disability-adjusted life years (DALYs) for 291 diseases and injuries in 21 regions, 1990-2010: a systematic analysis for the Global Burden of Disease Study 2010. Lancet 380, 2197–2223. 10.1016/S0140-6736(12)61689-4 23245608

[B27] O'Donnell M. J. Xavier D. Liu L. Zhang H. Chin S. L. Rao-Melacini P. . (2010). Risk factors for ischaemic and intracerebral haemorrhagic stroke in 22 countries (the INTERSTROKE study): a case-control study. Lancet 376, 112–123. 10.1016/S0140-6736(10)60834-3 20561675

[B28] Perry D. C. Sturm V. E. Peterson M. J. Pieper C. F. Bullock T. Boeve B. F. . (2016). Association of traumatic brain injury with subsequent neurological and psychiatric disease: a meta-analysis. J. Neurosurg. 124, 511–526. 10.3171/2015.2.JNS14503 26315003 PMC4751029

[B29] Spahn D. R. Bouillon B. Cerny V. Duranteau J. Filipescu D. Hunt B. J. . (2019). The European guideline on management of major bleeding and coagulopathy following trauma: fifth edition. Crit. Care 23:98. 10.1186/s13054-019-2347-3 30917843 PMC6436241

[B30] Stang A. (2010). Critical evaluation of the Newcastle-Ottawa scale for the assessment of the quality of nonrandomized studies in meta-analyses. Eur. J. Epidemiol. 25, 603–605. 10.1007/s10654-010-9491-z 20652370

[B31] Steinman J. Cahill L. S. Koletar M. M. Stefanovic B. Sled J. G. (2019). Acute and chronic stage adaptations of vascular architecture and cerebral blood flow in a mouse model of TBI. Neuroimage 202:116101. 10.1016/j.neuroimage.2019.116101 31425794

[B32] Stocchetti N. Zanier E. R. (2016). Chronic impact of traumatic brain injury on outcome and quality of life: a narrative review. Crit. Care 20, 148–148. 10.1186/s13054-016-1318-1 27323708 PMC4915181

[B33] Stroup D. F. Berlin J. A. Morton S. C. Olkin I. Williamson G. D. Rennie D. . (2000). Meta-analysis of observational studies in epidemiology: a proposal for reporting. Meta-analysis Of Observational Studies in Epidemiology (MOOSE) group. JAMA 283, 2008–2012. 10.1001/jama.283.15.2008 10789670

[B34] Taylor L. G. Panucci G. Mosholder A. D. Toh S. Huang T. Y. (2019). Antipsychotic use and stroke: a retrospective comparative study in a non-elderly population. J. Clin. Psychiatry 80:18m12636. 10.4088/JCP.18m12636 31163104

[B35] Thomalla G. Boutitie F. Fiebach J. B. Simonsen C. Z. Nighoghossian N. Pedraza S. . (2017). Stroke with unknown time of symptom onset: baseline clinical and magnetic resonance imaging data of the first thousand patients in WAKE-UP (efficacy and safety of MRI-based thrombolysis in wake-up stroke: a randomized, doubleblind, placebo-controlled trial). Stroke 48, 770–773. 10.1161/STROKEAHA.116.015233 28174327

[B36] Vaaramo K. Puljula J. Tetri S. Juvela S. Hillbom M. (2014). Predictors of new-onset seizures: a 10-year follow-up of head trauma subjects with and without traumatic brain injury. J. Neurol. Neurosurg. Psychiatry 85, 598–602. 10.1136/jnnp-2012-304457 23761917

[B37] VanItallie T. B. (2019). Traumatic brain injury (TBI) in collision sports: possible mechanisms of transformation into chronic traumatic encephalopathy (CTE). Metabolism 100:153943. 10.1016/j.metabol.2019.07.007 31610856

[B38] Wang W. Jiang B. Sun H. Ru X. Sun D. Wang L. . (2017). Prevalence, incidence, and mortality of stroke in China: results from a nationwide population-based survey of 480 687 adults. Circulation 135, 759–771. 10.1161/CIRCULATIONAHA.116.025250 28052979

[B39] Whiting D. DiNardo J. A. (2014). TEG and ROTEM: technology and clinical applications. Am. J. Hematol. 89, 228–232. 10.1002/ajh.23599 24123050

[B40] Wilson L. Stewart W. Dams-O'Connor K. Diaz-Arrastia R. Horton L. Menon D. K. . (2017). The chronic and evolving neurological consequences of traumatic brain injury. Lancet Neurol. 16, 813–825. 10.1016/S1474-4422(17)30279-X 28920887 PMC9336016

