# Supplementary figures and images for: Traumatic Brain Injury Is Associated With Both Hemorrhagic Stroke and Ischemic Stroke: A Systematic Review and Meta-Analysis
Source: Front Neurosci. 2022 Feb 10;16:814684. doi: 10.3389/fnins.2022.814684 (PMC8867812; doi:10.3389/fnins.2022.814684)

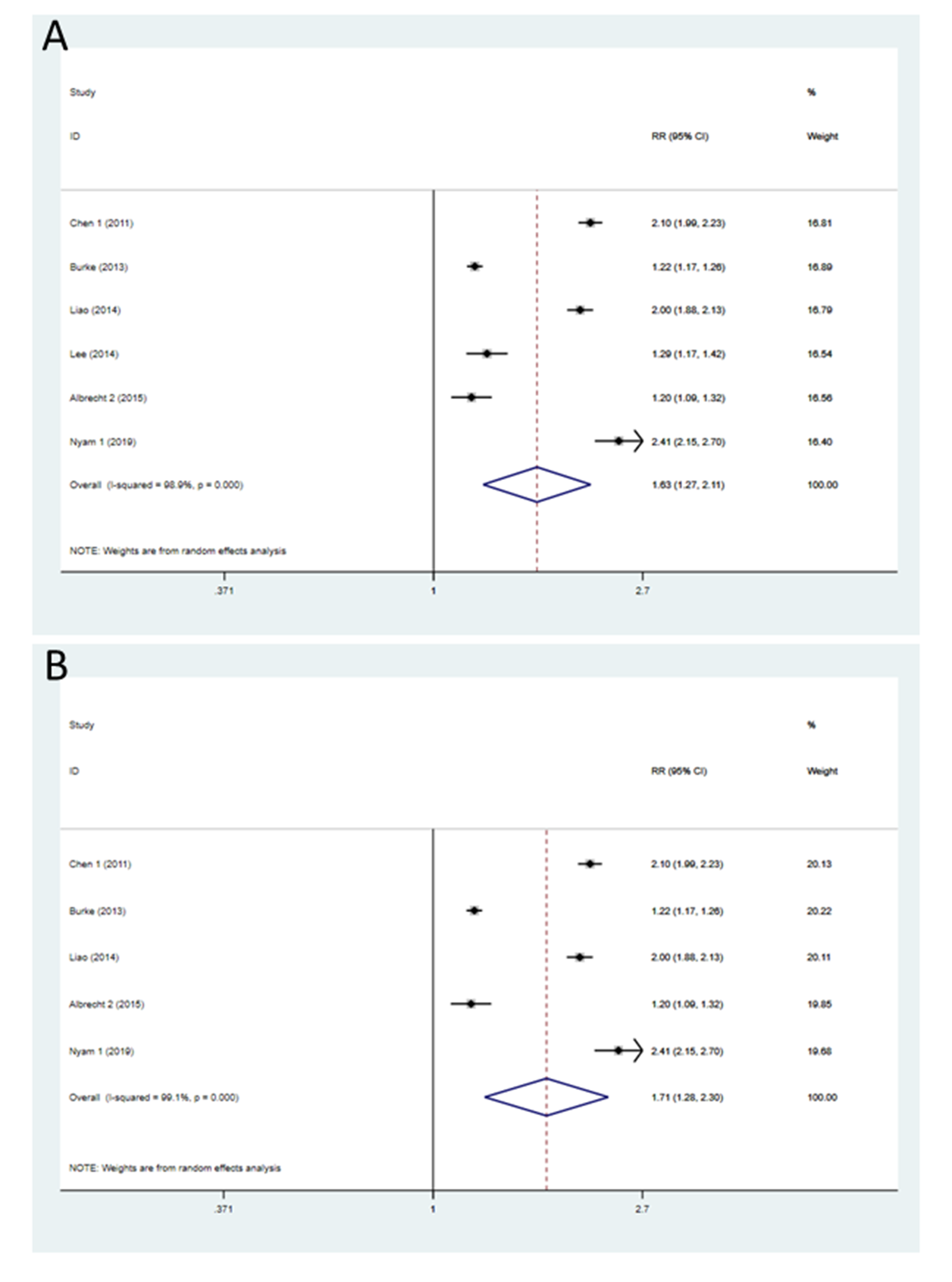

Supplement: Supplementary Figure 1 — Sensitivity analysis on gender. (A) Forest plots of the association between traumatic brain injury and future stroke in all included studies. (B) Forest plots of the association between traumatic brain injury and future stroke after excluding the study that had an unbalanced distribution of gender between TBI and control groups. Diamonds represent the pooled estimates, and the horizontal lines represent the 95% confidence intervals. RR, relative risk; CI, confidence interval. [file Image_1.TIF]

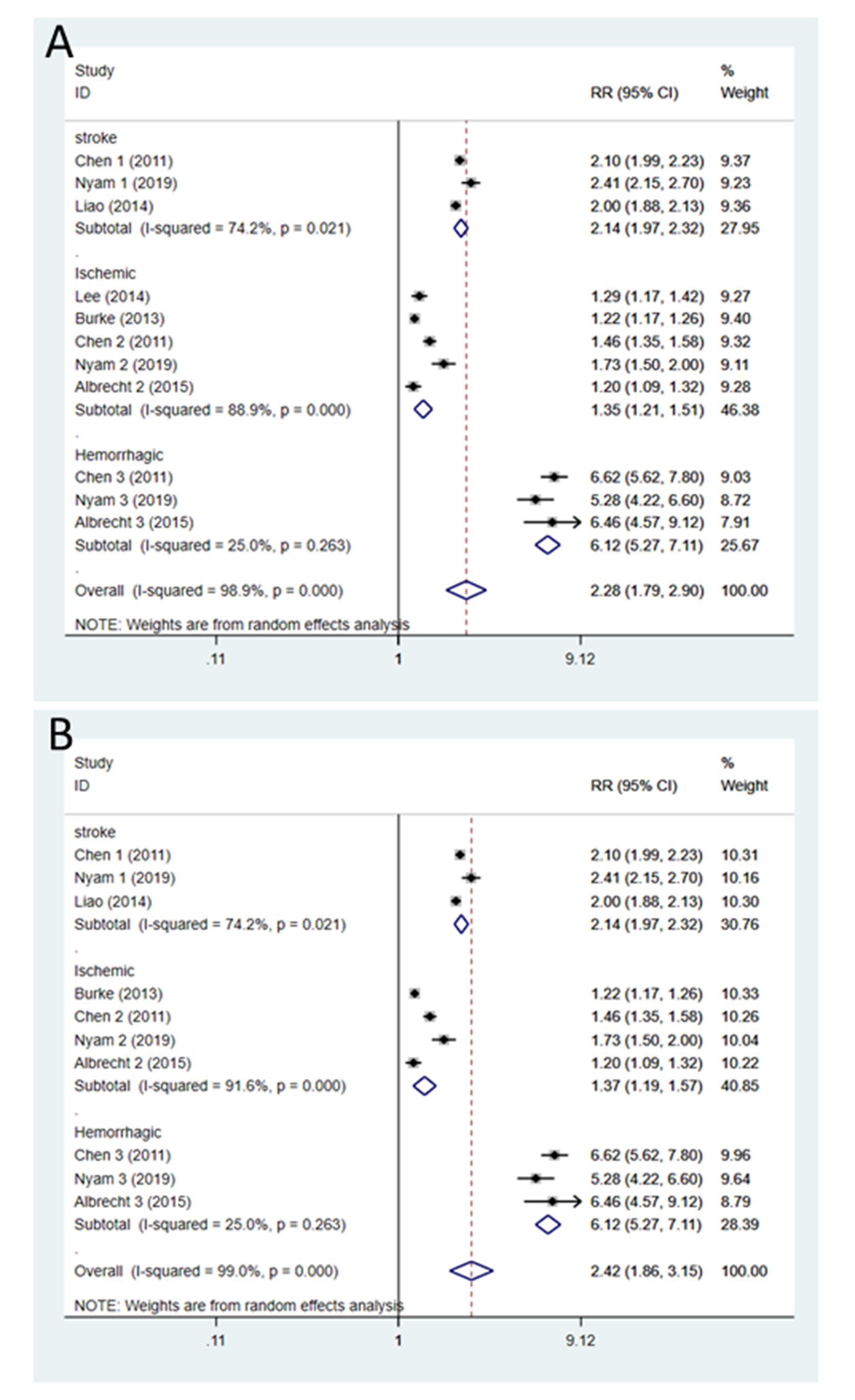

Supplement: Supplementary Figure 2 — Gender sensitivity analysis in subgroup analysis. (A) Forest plots of subgroup analysis of the association between traumatic brain injury and all types of stroke in all included studies. (B) Forest plots of subgroup analysis of the association between traumatic brain injury and all types of stroke after excluding the study that had an unbalanced distribution of gender between TBI and control groups. Diamonds represent the pooled estimates, and the horizontal lines represent the 95% confidence intervals. RR, relative risk; CI, confidence interval. [file Image_2.TIF]
